# Personalized multicomponent exercise programs using smartphone technology among older people: protocol for a randomized controlled trial

**DOI:** 10.1186/s12877-021-02559-2

**Published:** 2021-10-26

**Authors:** Yael Netz, Esther Argov, Ziv Yekutieli, Moshe Ayalon, Keren Tchelet, David Ben-Sira, Yihya Amar, Jeremy M. Jacobs

**Affiliations:** 1 The Academic College at Wingate, 4209200 Netanya, Israel; 2 Montfort Brain Monitor LTD, Binyamina, Israel; 3 Faculty of Medicine, Department of Geriatric Rehabilitation, Hadassah Medical Center, Hebrew University of Jerusalem, Jerusalem, Israel

**Keywords:** Exercise prescription, Individualized program, Remote assessment, Artificial intelligence, Motor fitness

## Abstract

**Background:**

Optimal application of the recently updated World Health Organization (WHO) guidelines for exercise in advanced age necessitates an accurate adjustment for the age-related increasing variability in biological age and fitness levels, alongside detailed recommendations across a range of motor fitness components, including balance, strength, and flexibility. We previously developed and validated a novel tool, designed to both remotely assess these fitness components, and subsequently deliver a personalized exercise program via smartphone. We describe the design of a prospective randomized control trial, comparing the effectiveness of the remotely delivered personalized multicomponent exercise program to either WHO exercise guidelines or no intervention.

**Methods:**

Participants (*n* = 300) are community dwelling, healthy, functionally independent, cognitively intact volunteers aged ≥65 at low risk for serious fall injuries, assigned using permuted block randomization (age/gender) to intervention, active-control, or control group. The intervention is an 8-week program including individually tailored exercises for upper/lower body, flexibility, strength, and balance (dynamic, static, vestibular); active-controls receive exercising counselling according to WHO guidelines; controls receive no guidance. Primary outcome is participant fitness level, operationalized as 42 digital markers generated from 10 motor fitness measures (balance, strength, flexibility); measured at baseline, mid-trial (4-weeks), trial-end (8-weeks), and follow-up (12-weeks). Target sample size is 300 participants to provide 99% power for moderate and high effect sizes (Cohen’s f = 0.25, 0.40 respectively).

**Discussion:**

The study will help understand the value of individualized motor fitness assessment used to generate personalized multicomponent exercise programs, delivered remotely among older adults.

**Trial registration:**

ClinicalTrials.gov Identifier: NCT04181983

**Supplementary Information:**

The online version contains supplementary material available at 10.1186/s12877-021-02559-2.

## Background

In 2020 the World Health Organization (WHO) published the new evidence-based guidelines for exercise among adults aged ≥65, comprising 150–300 min of moderate-intensity, or 75–150 min of vigorous-intensity physical activity, per week, combined with ≥three times a week of multicomponent physical activity that emphasizes functional balance and strength training at moderate or greater intensity [1]. In order to optimize the benefits of these exercise guidelines across the wide and varied target population of older people, two major challenges must be addressed.

Firstly, the widening gap between chronological and biological age, which typifies advancing age, raises into question the benefits of general guidelines for a certain chronological age. The growing variability between biological and chronological age is well recognized in aging research, and highlights the dangers and inexactitude involved in chronological age determined standards and reference ranges [2]. For example, modeling the relationship between numerus biological markers and an individual’s chronological age raises questions about the nature of aging [2], as witnessed by the differential trajectories of age-related decline experienced by individuals with the same chronological age. Thus biological age should be assessed distinctly from chronological age [3].

Furthermore, the gap between biological and chronological age widens with advancing age across a multitude of molecular and physiological systems [3] as well as performance measures [4]. This gap raises the question whether “one-size-fits-all” strategy in exercise recommendations for older adults is effective. This question is even more relevant in light of the rapid growth of the field of precision personalized medicine, enabled by artificial intelligence (AI). According to Johnson et al., [5], current research which integrates AI into precision medicine, is driving towards a highly personalized medical diagnostic and therapeutic information. While personalized medicine refers primarily to diagnostics and therapeutics for disease treatment, it also emphasizes the prevention and risks prediction aspects as well as the health or wellbeing of every person [6, 7].

A second challenge is the imprecise information provided in the general guidelines regarding fitness components related to the musculoskeletal (movement) system as opposed to the cardiovascular system. While the guidelines for aerobic exercise (aimed at enhancing the cardiovascular system) are specific in terms of optimal dose, intensity or duration, the guidelines remain vague for strength, balance and flexibility (movement system). One reason may be attributed to difficulties in accurate measurement, required for an accurate assessment of the level of fitness in skeletal muscle performance or postural stability in non-laboratory settings. Another reason is the priority given by researchers to energy expenditure, mainly aerobic exercise, in promoting health (e.g. [8–10]). The promotion of aerobic activity is often at the expense of under emphasizing the fitness of the motor system, including balance, strength and flexibility- all of which are critically important in preventing falls, improving the musculo-skeletal performance, and range of motion ([11–13] respectively) in advanced age. It is therefore not surprising that technological innovations to promote and monitor physical activity on an individual basis, such as fitness trackers and mobile phone applications, which usually measure accumulation of repetitive movements, such as the number of steps or movements of legs or arms, are designed primarily to provide information on energy expenditure equivalent to cardiovascular (aerobic) exercise (e.g. [14–16]). Importantly, these technological innovations are typically designed to attract mainly young adults.

Recognizing the importance of addressing these challenges in order to promote optimal exercise among people over aged ≥65, the Israeli Ministry of Innovation, Science and Technology awarded a grant to develop a novel approach towards a personalized exercise program based on individualized assessment of multiple movement components in healthy older adults. Obviating the need for detailed assessment either in a laboratory or by trained professionals, we proposed to develop a novel tool able to remotely assess balance, flexibility, and strength using smartphone sensors (i.e. accelerometer and gyroscope). Based upon data from this multicomponent assessment, the study tool was then designed to subsequently deliver personalized exercise programs tailored to address these movement components via the smartphone.

We recently described the design, development, validation, and results from the pilot study [17], which confirm the proof of concept and feasibility of the study tool. Briefly, an interdisciplinary panel of experts chose the motor components to be included in the remote fitness assessment, and the standard movement performance tests to best assess them. These were incorporated into a smartphone application, designed to present a user-friendly fitness test protocol on the smartphone, including audiovisual cues and instructions, whilst simultaneously measuring and recording the test result data. In order to deliver the personalized exercise program via the smartphone, an assorted collection of exercises graded for different levels of movement abilities and specifically designed to meet motor fitness requirements for older adults, was developed, photographed, filmed, and uploaded to the new application.

We enrolled 52 volunteers in the pilot study. Based on remote smartphone assessment of their fitness level at baseline, participants were assigned a 6 week personalized exercise program. Each participant underwent the smartphone fitness assessment at baseline and again at 6 weeks after completion of the personalized exercise program. The results of remote smartphone fitness assessment were validated against “gold standard” fitness testing of the same components among the pilot study participants, who were also assessed at baseline and after 6 weeks in the study sports laboratory. The results of the pilot study provided proof of concept, with both improved fitness and good adherence confirming the benefits and feasibility of remote fitness assessment for guiding home personalized exercise programs among healthy adults aged > 65. Artificial intelligence (AI) was used to analyze data collected in the pilot study (from remote fitness testing before and after the different exercise components within the personal exercise plans) in order to further refine the study tool. As the study data base continues to enlarge, with subsequent big-data collection from additional participants, it is planned that AI will be integrated into the study tool to constantly refine and optimize the match between a participant’s fitness assessment and their most suitable personalized exercise program.

Having successfully completed the pilot study, we present the protocol for the next study stage, which plans to conduct a prospective randomized controlled trial using the study tool among healthy older people. Specifically, the study objectives are to investigate the effectiveness of a remotely delivered personalized 8-week multicomponent exercise program based upon remote individual fitness assessment, compared either to the updated WHO guidelines (active-control) or to no intervention (control). We hypothesize that participants in the personalized experimental group will show greatest motor improvement in balance, strength, and flexibility than participants with similar baseline fitness profiles in the active-control, with least changes observed among the control group.

## Methods/design

### Design and participants

The study is an interventional, prospective randomized control trial, comparing the study intervention to an active and passive control. Participants are healthy community dwelling volunteers aged ≥65 years, recruited using flyers and lectures by the study principal investigators at local elderly clubs, day centers, and independent living facilities. The study setting will be community based, either at home, local community centers or independent living facilities.

### Eligibility, inclusion and exclusion criteria

Healthy volunteers aged ≥65 years, males and females are eligible, who for inclusion must meet all the following criteria: 1) home or independent living; 2) fluent Hebrew speakers; 3) able to walk independently without help from another person; 4) functionally independent in dressing, toileting, grooming, washing, eating; 5) a smartphone user.

Exclusion criteria are ≥1 positive from the following: 1) Cognitive decline < 3/5 on Mini-Cog Score [18]; 2) Any hospitalization (> 24 h) or at least one Emergency Room referral in previous 12 months; due to unstable heart disease (congestive heart disease/rhythm disorder/ischemic heart disease/valvular heart disease) or neurological disease including balance or dizziness (cerebrovascular disease, vestibular disease, progressive neurological disease affecting gait or balance). (Information by self-report on direct questioning); 3) High risk of falling, as assessed by any positive answer to one of the 3 validated questions previously used in community-based exercise intervention and fall prevention studies [19]: a) Have you fallen over twice or more in the last year?; b) Have you fallen and hurt yourself in the last year?; c) Are you afraid that you might fall because of balance or walking problems?; 4) Unwilling to provide consent.

All participants will undergo assessment by the study physician in order to assess eligibility. The study physician will also gain informed signed consent, whereupon preliminary background data will be collected by trained research assistants. All participants who during the study trial experience an event listed in the exclusion criteria will be asked to discontinue the trial. Similarly, participants will be free to leave the trial at any time. In addition, participants will be asked to refrain from initiating new additional exercise programs during the study period.

#### Assignment of intervention

Participants will be randomly assigned to three interventions groups 1) the experimental group (personalized exercise using study-app); 2) an active control (counseling concerning standard WHO guidelines); and 3) a control group (no intervention). We will enroll 300 participants.

### Intervention

**Fig. 1:**
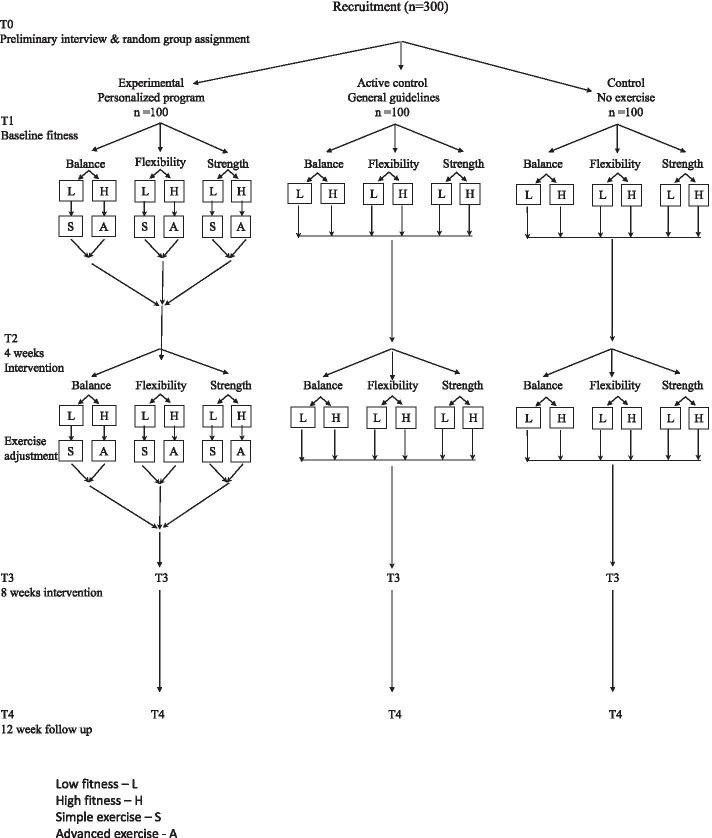
Flow chart of study design

As shown in in the study flow chart (Fig. 1), the intervention will last 8 weeks. Following informed consent forms and collection of preliminary demographic and clinical data (T0), all participants will undergo fitness assessment by the test app before the intervention (T1), in which their fitness (Low or High) will be determined. They will then be re-assessed after 4 weeks (T2), after 8 weeks (T3), and after 12 weeks (follow-up T4). The testers will be well trained research assistants who will use their own mobile phone for the testing.

### Intervention groups

**Fig. 2:**
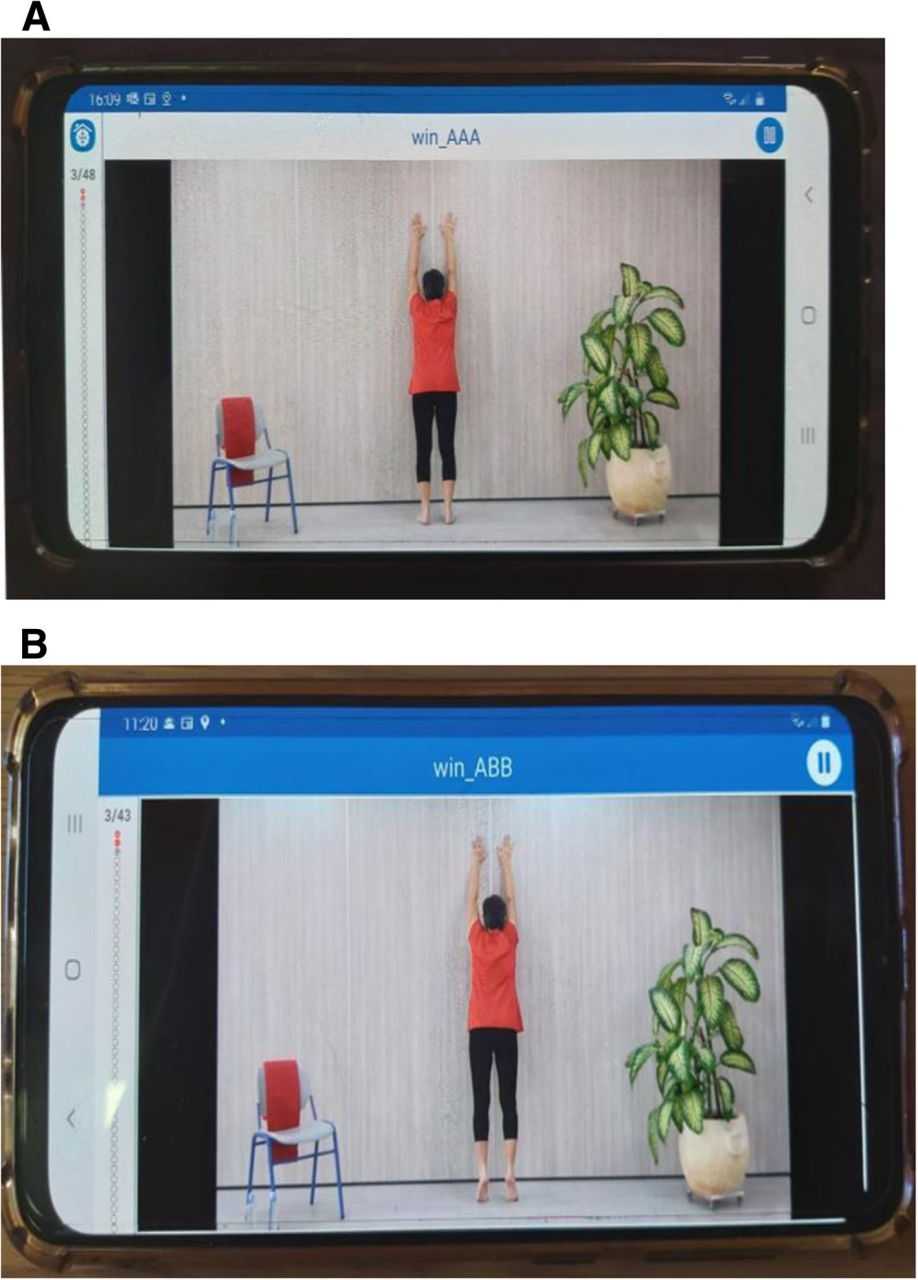
An example of exercise for arms flexibility for level A and B as displayed on phone

1. ***The experimental group (n = 100):*** participants will receive their personalized exercise program based upon the assessment of their performance level in each of the following fitness component: balance, flexibility, strength. Following the pilot study [17], and according to evidence supporting the advantages of exercising > 3 times/week [1], they will be instructed to exercise five times/week. Their videoed exercise program will be delivered to their personal mobile phone. An example of exercise as displayed on the mobile phone is presented in Fig. 2. The exercises are grouped into three target categories: 1) balance and lower body exercises, 2) upper body flexibility exercises, and 3) upper body strength exercises. Each exercise was coded according to target categories, and two levels of difficulty based on two levels of fitness: A (simple level) and B (advanced). Examples of typical exercises for each of the three motor components, for both levels of difficulty are demonstrated in our pilot study [17].
2. ***An active control group (n = 100):*** participants will be asked, on individual basis, to exercise according to the official general guidelines published by the World Health Organization [1]. More specifically, they will be asked: 1) To perform leisure type aerobic exercise for 150-300 min or intensive exercise for 75-150 min. Per week. Examples such as walking, jogging, riding bicycle will be given; 2) In addition, they will be asked to perform ≥three times a week multicomponent physical activity that emphasizes functional balance and strength training at moderate or greater intensity. They will get the following examples of balance exercises: standing on toes, one leg-stance, walking while lifting knee, walking backwards, side walk while bending and extending knees. Examples of strength exercise: in standing position - lifting leg to side (abduction), extending straight leg backwards, lifting straight leg forward, sit and stand, standing against wall: pushing body with hands to wall, lifting arms to side – possibly with dumbbells.
3. ***A control group (n = 100***): participants will be advised to continue their normal routine, and if interested, will be able use the smart app exercise program after the completion of the study.

### Matching fitness level with exercise prescription (artificial intelligence)

**Fig. 3:**
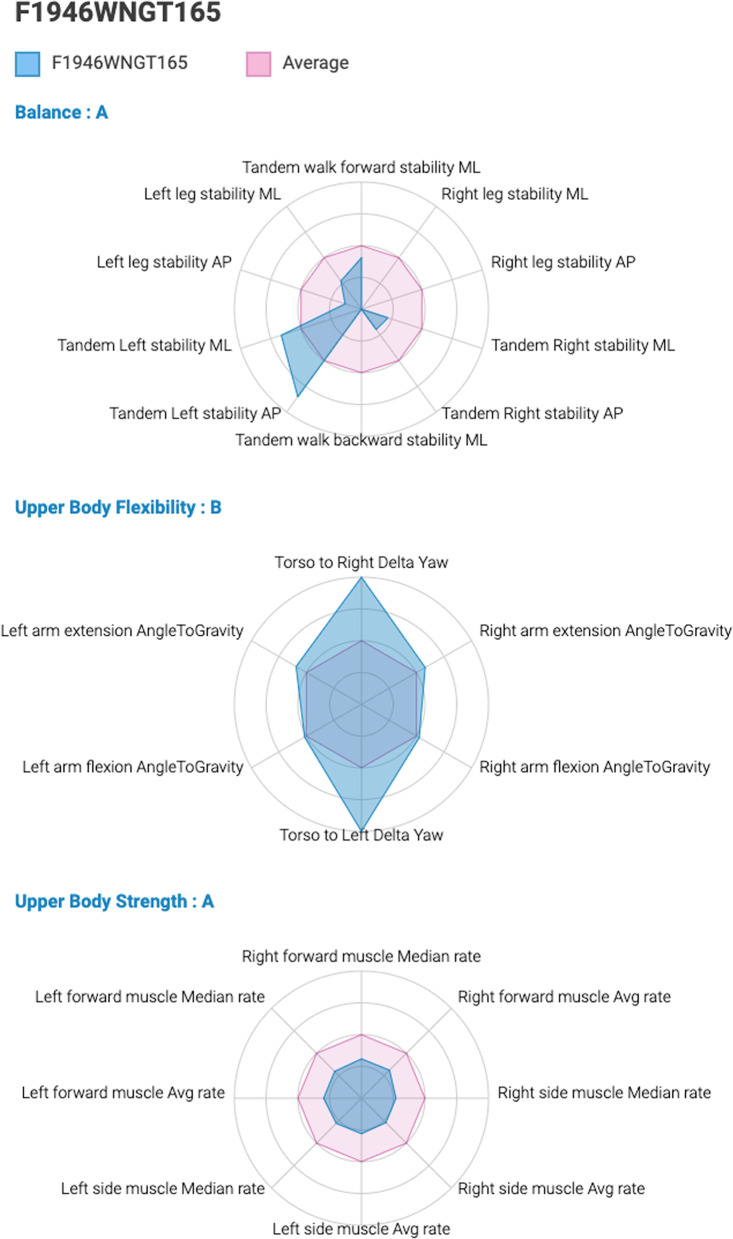
An example of a study participant’s unique fitness profile for balance, flexibility and strength

The data collected in the pilot study, served as a baseline for big-data collection and for subsequent use of artificial intelligence (AI). Based on this preliminary data collection and the AI, the system will determine the level of fitness (Low or High) of participants in the present study, and subsequently the level of difficulty of exercise to be assigned (A or B) for each fitness component. Figure 3 demonstrates an example of a study participant’s unique fitness profile based upon the digital markers, graphically displayed alongside the average profile for the complete study sample. The prescribed exercise, generated automatically following the baseline testing session (T1), will be directly delivered, via the mobile phone, to the participants of the personalized exercise (experimental) group, and adapted based on the scores of T2, if needed (see flow chart in Fig. 1). Prescribed exercise will be delivered to the control groups, upon request, after the termination of the intervention. As new data is obtained, the ability of the program to generate personalized exercise program will be further refined.

### Strategies to improve and monitor adherence

All participants from the experimental and active control groups will receive personal tutoring regarding his/her exercise program – personalized for the experimental group and general guidelines for the active control group. Participants in the experimental group will get specific guidance how to use their phone for watching their personalized program, and practice accordingly. In addition, they will get a small stand to place the phone in a viewable spot while exercising. Adherence to the programs will be monitored by a) Weekly phone calls to all participants. Participants in the experimental and active control groups will be inquired about difficulties or any comments they have regarding their exercise routine. In T2, T3 and T4 (every 4 weeks) they will be asked to report the average number of exercise sessions spent weekly in the previous 4 weeks. The passive control group will be informed in which week they are in the study, and when is the next fitness test due to occur.; b). The number of minutes spent using the study-app for exercising in the experimental group (the personalized exercise group) will be automatically recorded each time the smart-app exercise program is operated by participants in the intervention group.

### Guidelines for safety

Participants in all groups will be instructed how to act in case of any medical problem arising during the study (see ‘Ethics approval and consent to participate’ in administrative information). Specifically, the experimental (personalized exercise) group will be asked to watch two films delivered to their mobile phone prior to starting the exercise program, participants (see [17]), which will include the following safety rules: “wear comfortable clothes”, “exercise bare foot or wear non-slip socks”, “find a quiet room at home with a place to put an exercise mattress, with an empty wall, and importantly – with a stable chair with back rest”, “don’t exercise if you have pains (greater than 3 on a 1-10 scale)”, “in knees and hands position you can put a mattress under your knees”, “block incoming calls to your phone while you use it for exercising”, “full safety comes first”, “if you feel unsafe, skip the exercise, or stop the training session” “stop if you feel dizzy”. In addition, since some exercises are performed on a mattress, a short film will be shown demonstrating how to get down and up from mattress. The active-control (general guidelines) group will be advised to wear comfortable clothes for exercising, to use comfortable tennis shoes for the aerobic exercise, and tennis shoes or bare foot for the strength and balance exercise, to take safety precautions for fall prevention, and to stop exercising if feels unsafe, or if has pain. Comprehensive insurance will cover the possibility of any medical complications incurred during the study, and the potential need for compensation.

## Measurements

### Remote fitness assessment tool

**Fig. 4:**
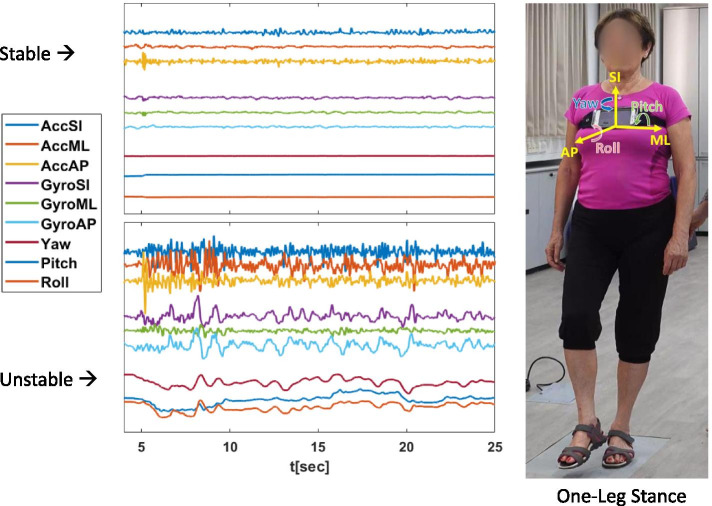
Graphical demonstration of raw data generated by smartphone accelerometer (Acc) and gyroscope (Gyro) for leg-stance (one of the authors is the demonstrator). Note. ML – Medio-lateral, from side to side; AP – Anterior-Posterior, forward and backward; SI – Superior-inferior, − up and down

We will use standard movement performance tests selected by experts and incorporated into the smartphone application (EncephaLog™) developed by Montfort Brain Monitor LTD, utilizing the standard smartphone sensors - accelerometers and gyroscopes. The assessment will include 10 performance tests, operationalized to generate a total of 42 digital markers (see Table 1 and Additional file 1). Briefly, the 10 performance tests assess the following fitness components: static balance, dynamic balance, leg strength, upper body flexibility, and upper extremities strength. Raw data will be uploaded to the remote pooled databank. An example showing graphical demonstration of the raw data collection for the “one-leg stance” is presented in Fig. 4.

**Table 1:** Outcome measures

Fitness Components	Tests	Digital Markers	Good performance
Static balance	^a^One-leg stance 10 s.	For each test: average acceleration (m/sec^2^) and angular velocity (radians/sec), in different directions.	*Lower scores – lower sway*
	^a^Tandem stance 20 s.		
Dynamic balance	Tandem walk: 10 steps forwards		
	Tandem walk: 10 steps backwards		
Legs strength	Sit-to-stand 10 rep.	Total time (sec.) and average duration for each rep.	*Shorter duration*
Upper body flexibility	^a^Torso rotation	Angle	*Increased angle - a greater range of motion*
	^a^Arm flexion		
	^a^Arm extension		
Upper extremities strength	^ab^Lifting arm forward 20 rep.	Average duration (sec.)	*Shorter duration*
	^ab^Lifting arm to side 20 rep.		

^a^For left and right separately

^b^with 0.5 kg for women and 1 kg for men

### Additional data

Preliminary background data, collected at interview at T0 will include: Sociodemographic data; Self-reported medical diagnoses and health related variables; Frailty Index [20]; Habitual physical activity (average time spent weekly for aerobic/non-aerobic exercise, daily physical activities, and sedentary time); Mini-Cog test validated Hebrew version [18]; and Geriatric depression score (short 5 item validated Hebrew version) [21].

In addition, the following data will be collected at throughout the study at T1-T4: A modified version of the Flanker Task [22] assessing information processing in visual search (cognition), and the Profiles of Mood State (POMS) [23] validated Hebrew version of the scale [24], consisting of positive and negative feelings felt in previous week.

The sociodemographic, physical activity mood and cognition data be collected by the research assistants, and all other data by the study physicians. All information will be uploaded directly to the mobile phone of the testers, and automatically remotely delivered to the platform.

#### Data Collection and Management

Each participant will be given a confidential study number, with identification record kept separately from all data by the principal investigators (PIs). Data from smartphone collected throughout the study will be uploaded remotely via the study-app platform (by Montfort Brain Monitor), de-identified using the confidential study number.

Data will be stored and analyzed using Microsoft Azure - authentication and authorization with Microsoft Azure Active Directory (Single security token is valid for 60 min). Participant coding on Montfort’s side is pseudonymized, only the PIs know the participants’ personal details. The data is sent to Montfort servers via Hypertext Transfer Protocol Secure (HTTPS), with 2048 bits Secure Sockets Layer (SSL). Local device data is deleted within 2 days after successful upload. Uploaded data is stored on the server for a minimum of 2 years. Data is accessible by Montfort and by 3 research team members at the Wingate College.

### Primary outcomes

The primary outcome measures are the changes in the following motor components, all of which are included in the remote fitness assessment (see Table 1 and Additional file 1): static balance (postural control in standing), dynamic balance (postural control while moving), strength (muscle endurance) of upper and lower extremities, and flexibility (range of motion in upper and lower body). All outcomes will be measured among all participants at baseline (T1), at 4 weeks (mid-intervention) (T2), at 8 weeks (end of intervention) (T3), at 12 weeks (one-month post intervention) (T4).

### Secondary outcomes

Adherence to the exercise programs and changes in feelings and cognition will be compared between the different study groups. In addition, age (65-74 vs 75+) and gender differences in the changes in the primary outcomes will be examined, as well as levels of fitness (Low vs High).

## Statistical methods

### Randomization

Following consent, participants will be assigned a unique confidential study number. They will then be randomly assigned to the three intervention groups using R [25]. We will use permuted block randomization for the following 4 groups: young women and young men (age 65-74), and old women and old men (age 75+).

### Sample size and power analysis

Statistical power was calculated using G * Power [26]. Based on the analysis, 100 participants in each of the 3 treatment groups (experimental, active control, control) enable detecting group differences in statistical power of 86% for a small effect size (Cohen’s f = .15), and in statistical power of 99% for a moderate and high effect sizes (Cohen’s f = .25, .40 respectively). In addition, the statistical power for detecting differences between repeated measures, and in the interaction between groups in the repeated measures is higher than 99% in the moderate effect size as well as in the high effect size.

#### Data analysis

The following 2-ways ANOVAs with repeated measures (3 treatment groups X 4 times measurements – T1-T4) will be conducted for each digital marker as follows:

- For all participants
- Separately for each of the following four subgroups: young women and young men (age 65-74), and old women and old men (age 75+).
- Separately for each of the two levels of fitness (Low and High) for each component. For example: low level of balance performance in the experimental group (personalized) compared to low level of balance in the control groups.
  Bonferroni will be used for post-hoc analyses, and False Discovery Rate [27] will be used to account for multiple comparisons.

## Discussion

This approach provides new potential solutions to combat the decline in physical activity which accompanies advancing age [28], and which has recently witnessed further exacerbation due to social isolation caused by the COVID-19 pandemic [29]. Furthermore, this model takes into consideration the increasing variability in fitness parameters, which like numerous other biological markers, exhibit a widening inter-individual variability with advancing age [4].

The current study serves to strengthen the proof of concept which underlies our study tool, combining both personalized exercise programs and individualized fitness assessment together with smartphone technology. The innovative technology will create a validated digital app which is dedicated to providing assessment of motor fitness along with a dynamic exercise program individually tailored based on the older person’s current ability level. If the study is successful, the results will serve to deepen contemporary approach to personalized exercise programs among the rapidly growing older population, and may serve as the baseline for numerous variations of personalized exercise programs. Furthermore, programs can be streamlined to incorporate variations based on the person’s movement preferences, and targeted at specific populations such as people confined to wheelchairs, those with mild cognitive limitations, as well as pre-frail or even frail individuals. Given the global pandemic of physical inactivity, and based on trend towards ever more personalized medicine, this innovative concept of a personalized exercise program may be further developed for use in all ages and at all functioning levels.

## Limitations

1. In order to avoid small comparison groups which may create statistical difficulties, we will apply only two levels of difficulty for each of the three movement components (eight lessons). As data from testing accumulates, we will apply three or more levels of difficulty for each component across different groups, enabling providing more accurate individualized exercise programs.
2. Although the photos (videos) of the exercises, which have been produced by a professional photographer, are very clear, and presented on a stabilized (on a stand) mobile phone, the display of the mobile phone is nonetheless rather small. Our plan is to find a simple technological solution for transferring the videoed exercise programs to big screens (e.g. Television screen).
3. There is a built-in conflict between the concept of personalized exercise tailored according to one’s needs, and performed at home at any convenient time, and the social benefits attributed to group exercise. To overcome this limitation, our future plan is to create, in addition to individual exercise programs, group activity programs where each person is performing his/her personal program.
4. Conceptually, personal preferences should be considered in individually tailored exercise program. Due to methodological limitations (keeping the study well-controlled), we will not incorporate the person’s movement preferences into the objective levels of fitness in this study.

## Supplementary Information

**Additional file 1.** A description of the 10 tests (42 digital markers).

## Data Availability

The datasets generated during and/or analyzed during the study will be available from the corresponding author on reasonable request.

## Abbreviations

WHO: World Health Organization
AI: Artificial intelligence
T0-T4: Time 0 – time 4
Mini-Cog test: Mini cognition test
Acc: Accelerometer
Gyro: Gyroscope
ML: Medio-lateral
AP: Anterior-posterior
SI: Superior-inferior
POMS: Profiles of Mood States
PI: Principal investigator
ANOVA: Analysis of variance
HTTPS: Hypertext Transfer Protocol Secure
SSL: Secure Sockets Layer
