# Supplementary material for: Personalized multicomponent exercise programs using smartphone technology among older people: protocol for a randomized controlled trial
Source: BMC Geriatr. 2021 Oct 26;21:605. doi: 10.1186/s12877-021-02559-2 (PMC8547559; doi:10.1186/s12877-021-02559-2)
Supplement: Supplementary file 1 — Additional file 1. A description of the 10 tests (42 digital markers). [file 12877_2021_2559_MOESM1_ESM.docx]

**Additional file 1**

**A description of the 10 tests (42 digital markers)**

***Balance (postural sway)***

***Static balance*** – 4 tests

*One leg stance* (left and right) for a duration of 10 seconds, and *tandem stance* (left foot forward, and right foot forward) for a duration of 20 seconds.

***Dynamic balance*** *–* 2 tests

10 steps *tandem walk forward and backward*.

Five relevant digital markers (DMs) will assess the sway in each of the above 6 tests (n=30): average linear acceleration assessed in meter per second squared (m/sec^2^), which is generated from the accelerometer (Acc) in ML (medio-lateral) and AP (anterior-posterior) directions, and angular (radial) velocity assessed in radians per second (rad/sec), which is generated from the gyroscope (Gyro) in the ML, AP, and SI (superior-inferior) directions. Lower scores indicate less sway, thus better performance.

***Legs strength*:** *Sit-to-Stand* –10 repetitions. DMs (n=2): total time (seconds) and average duration for each repetition. Shorter duration indicates better performance.

***Upper body flexibility*** (each test will be performed twice and the score will be the average of the two)

*Torso rotation:* In a seated position, holding a ball between thighs and a stick on chest for stabilization: torso rotation as far as possible to the right and to the left. DMs (n=2): angle (peak pitch in the SI direction). Increased angle indicates a greater range of motion, thus better performance.

*Upper extremities flexibility*:

A*rm flexion*: In a seated position on an armless chair, back against the wall – lifting straight arm forward and up trying to reach the ear (right and left). DMs (n=2): angle between arm and the horizon (peak yaw in the AP direction). Increased angle indicates a greater range of motion, thus better performance.

A*rm extension*: In a standing position, face toward the wall, all front body against the wall – lifting straight arm backward as far as possible (right and left). DMs (n=2): angle between arm and the horizon (peak yaw in the AP direction). Increased angle indicates a greater range of motion, thus better performance.

***Upper extremities strength***

In a seated position on an armless chair, a weight of 0.5 kg for women and 1 kg for men attached to wrist:

*Lifting straight arm forward* to shoulder *height* as fast as possible 20 times (right and left arm), and *to the side* 20 times (right and left arm). DMs (n=4): average duration for the 20 repetitions measured in seconds. A shorter duration indicates better performance.
